# Inter-arm bone mass and size asymmetries in children tennis players are maturity status specific: a 9-month study on the effects of training time across pubertal change and somatic growth

**DOI:** 10.1007/s00421-024-05425-2

**Published:** 2024-02-28

**Authors:** Dimitria Palaiothodorou, George Vagenas

**Affiliations:** School of Physical Education and Sport Science, National and Kapodistrian University of Athens, Athens, Greece

**Keywords:** Growth, Children, Puberty, Bone mass, Bone size, Tennis, Asymmetries

## Abstract

**Purpose:**

Bone growth with exercise is best assessed by tennis-induced inter-arm asymmetries. Yet, the effects of training and maturation across puberty were unclear. This study explored arm bone growth across 9 months of training in 46 tennis players 7–14 years (25 boys, 21 girls).

**Methods:**

Bone mineral content (BMC) and bone area (BA) were measured from DXA scans. Pubertal status was assessed by Tanner stage (TS) and somatic growth by maturity offset (MO). Children were grouped as pre- (TS I–I), early (TS I–II), and mid/late pubertal (TS II–III).

**Results:**

Training time (TT) change in the three groups was 160–170, 190–230, and 200–220 h, respectively. Bone asymmetries were large in all groups (*d* > 0.8, *P* < 0.001): 5–18 g (9–21%) and 9–17 g (17–23%) in girls and boys, respectively, for BMC, and 5–15 cm^2^ (6–13%) and 9–15 cm^2^ (12–15%) in girls and boys (10–13%), respectively, for BA. BMC and BA change asymmetry peaked at pre-puberty in girls (56%, 46%) and at early puberty in boys (57%, 43%). Asymmetry gains varied with baseline asymmetry (41%) and change in TT (38%) and TS (17%) in BMC, and with baseline asymmetry (58%) and change in MO (17%) and TS (12%) in BA.

**Conclusion:**

All bone asymmetries were substantial. Tennis-induced bone gains were higher at pre- to early puberty in girls and at early to mid/late puberty in boys. Training enhanced mostly bone mass and maturity status enhanced mostly bone size; sex was not bone-change modeling impactful. Implications are discussed considering certain limitations.

## Introduction

The integrity of the bones (Burr et al. 1997) and the development of the skeleton (Bailey and Martin 1994) are both vital health properties that can be maximized with proper exercise. The extra strength gained by the active bones during childhood adds significant protection against adolescent bone fractures (Faulkner et al. 2006). The extension of this bone strength into adulthood helps older people to better deal with osteoporosis and related issues (Bachrach 2005; Khan et al. 2000). Yet, notable bone adaptations arise merely under dynamic rather than static mechanical conditions (Turner 1998), while vitamins, genes, and hormones coregulate the activity of bone cells (Frost 1987, 2000). Growth factors and sex steroids determine the rate and degree of mineralization and the onset of puberty (Mauras et al. 1996), conditional on the genetic regulation of somatic stature (Beunen et al. 2000) and peak bone mass (Mølgaard et al. 2001). Hence, the assessment of bone change with exercise requires control for the confounding effects of heredity, hormones, nutrition, and lifestyle (Calbet et al. 1998; Ilich and Kerstetter 2000). Assuming no resultant bias from the offsetting effects of two-handed backhands (Ducher et al. 2005) and handedness (Krahl et al. 1994), tennis best provides the dynamic arm conditions and the control of these confounders, the effects of which are equal to both arms (Sanchis-Moysi et al. 2010b). Thus, the osteogenic effects of exercise are quantified by the asymmetry between the racket arm (RacArm) and the opposite arm (OppArm), the latter reflecting normal growth (Pirnay et al. 1987).

Tennis-induced osteogenesis varies with age, sex, and maturation, and relates merely to bone mass (Huddleston et al. 1980; Kannus et al. 1994) and bone size (Krahl et al. 1994; Maughan et al. 1986). This osteogenesis tends to be higher in early than late starters, in males than females (Haapasalo et al. 1996), and in humeral than radio-ulnar bones (Kontulainen et al. 1999). Yet, conditional on the measure and magnitude of training volume, it also tends to progress about quadratically between pre-, peri-, and post-pubertal girls (Bass et al. 2002) and boys (Ducher et al. 2009). Thus, the analogy between bone gain and exercise duration becomes maturity stage specific and differential between weekly hours and total training time. For instance, tennis-induced BMC and BA asymmetries increased from 12 and 8%, respectively, at pre-puberty when training was 2–4 h/week, to 22% and 16%, respectively, at peri-puberty when training tripled to 8–13 h/week (Sanchis-Moysi et al. 2010a). These gains increase from 11 and 7%, respectively, with 340 h of training (at pre-puberty) to 16–18% and 10–13%, respectively, when training doubles to 690 h (at peri-puberty) (Palaiothodorou et al. 2020). Hence, estimating how much bone mass and size is gained per certain exercise periods depends on changes within puberty. This growth period affects bone mass and strength enhancement (Ferrari et al. 2006) conditional on the strong relation between skeletal, sexual, and somatic growth (Marshall 1974).

So far only two tennis studies explored bone growth with puberty prospectively. Yet, both used puberty as a single period without considering sexual maturity and somatic growth. Ducher et al. (2011) examined female players 10–18 years and found that the humeral BMC and BA gains for the pre/peri-pubertal group improved from 18 and 9% to 21% and 18%, respectively, across 12 months of participation. Chapelle et al. (2023) examined players of both sexes 8–17 years and found no association between weekly training hours and “bone asymmetry development” before and after 2 years of participation; BMC asymmetry did not change from baseline (16–18%) to follow-up (18%), whereas the effects of sex per pubertal stage were untested. The results of these studies left space for further study. Thus, the present study aimed to examine children tennis players aged < 15 years before and after 9 months of participation, after controlling for sex and maturation according to Tanner stage (Baxter-Jones et al. 2005) and MO (Malina et al. 2006). Our objectives were to quantify the impact of training duration on BMC and BA, in pre-, early, and mid/late-pubertal girls and boys, to describe the age-progressing patterns of bone mass and size change from childhood to late puberty, and to model the combined effects of training time, sex, pubertal stage (TS), and MO (e.g., Morris et al. 1997).

## Methods

### Participant and training traits

Forty-six tennis players aged 7–14 years (25 boys, 21 girls) from regional tennis clubs participated in the study. They were at the pre- and competitive level, trained at least 4 h per week, and free of any illness or musculoskeletal condition during and at least 6 months prior to enrollment. The choice of weekly training of > 3 h/week is in line with review conclusions that “a notable osteogenic effect may be achieved with just 3 h of participation in sports” (Vicente-Rodríguez 2006). Their inclusion was agreed upon written consent of their parents. The study conformed with the Declaration of Helsinki guidelines and was approved by the University Ethics Committee and the Scientific Council of the Rehabilitation Center where testing took place.

A 9-month follow-up period was feasible for this sample of children. This was similar to periods of 8–12 months employed in previous relevant studies (Bradney et al. 1998; Ducher et al. 2011; Meyer et al. 2011). The training hours (h/w) for each successive week across follow-up were recorded to obtain total training time (TT) as the best proxy of training volume (e.g., Kannus et al. 1994). All children used one-handed forehands and two-handed backhands; 20 boys and 18 girls were right-handed. Racket arm (RacArm) was the arm used for forehand and service strokes. A priori power analysis indicated a sample of 19–14 children per group (i.e., 57–42 in total) to detect a bone asymmetry or a bone asymmetry change threshold of 5–10% (i.e., Cohen’s *d* ≈ 0.5) in 80% of the cases.

### Maturity status assessment

Sexual maturity was self-assessed with parental guidance as pubertal stage using Tanner’s scale for girls (Marshall and Tanner 1969) and boys (Marshall and Tanner 1970); a physician examination was unfeasible (Rasmussen et al. 2015). Children were classified as pre-, (TS I–I), early (TS I-II), mid- (TS II–III), or late pubertal (TS III–IV) (Rubin et al. 1993); the last two classes were merged to reduce design complexity as they share similar bone change patterns (Ducher et al. 2009).

Somatic growth was assessed by MO (Age-APHV) from sex-specific equations using height, weight, sitting height, leg length, and chronological age according to established procedures (Mirwald et al. 2002). There is always a need for enhanced accuracy of MO predictions in clinical, sports, and pediatric settings, and for the option to have these predictions without measuring sitting height (Moore et al. 2015). Therefore, APHV or MO offers an alternative control for somatic growth to classify differently maturing children (Baxter-Jones et al. 2005).

### Bone mass and size measurements

BMC and BA were measured from Dual X-Ray Absorptiometry (DXA) scans. This technique is preferred in pediatrics due to its speed, precision, and minimal radiation (Bachrach 2000). Thus, based on standard procedures (Libber et al. 2012), total body DXA scans (EnCore 2006, Lunar Prodigy, GE Medical System, Madison, WI USA) were obtained by a qualified radiographer. Total arm BMC (g) and BA (cm^2^) were calculated via regional bone analysis that secured inter-arm symmetry in separating line positioning (Bazzocchi et al. 2016).

Repeating the DXA test on part of the sample to check the reliability of body repositioning was unfeasible; we assumed this reliability (CV < 2%; ICC > 0.98) for children (Norland XR-26, Sievänen et al. 1993; GE Lunar Prodigy, Margulies et al. 2005). We thus checked intra-tester reliability in analyzing the scans of 15 children. BMC and BA values were highly reproducible (ICC > 0.99), with tiny variation (SEM < 0.81%) and almost perfect inter-arm symmetry (right: ICC > 0.99, SEM < 0.81%, left: ICC > 0.99, SEM < 0.91%). A similar low intra-tester reproducibility variation (RMS CV < 1.2%) has been found in tennis players aged 12–14 years (Ducher et al. 2006).

### Data processing and analysis

BMC and BA values were calculated for each arm. Asymmetry was then expressed as absolute (As = RacArm − OppArm) and percent (%As = 100 × As/OppArm). Descriptive statistics included means (± SD), range, and 95% CI. Change in BMC, BA, and asymmetry for boys and girls across follow-up was assessed as absolute (Δ), percent (%Δ), and standardized (*d*, Cohen 1988, pp 20–26). Change in BMC and BA asymmetry was regressed on the linear composite of sex, and change in TT, MO, and TS, with baseline asymmetry as covariate. TS change reflected a Tanner scale’s accuracy in separating pre-puberty from peri-puberty (Rasmussen et al. 2015) with this grossly approximating a binary signifying “no pubertal change” (merely TS I; pre-puberty, 21 children) vs “pubertal change” (mostly TS II-IV; early to late puberty, 25 children). Due to lack of previous similar model specifications, a practically useful threshold of extracted variance was not possible to be pre-estimated. Therefore, we relied on expert suggestions for 10–15 subjects per predictor (Maxwell 2000; Pedhazur 2006, pp 207–208); our 9–10 subjects/predictor ratio was close to the lower bound of this practical suggestion. Then, we used Shapley’s LMG value to estimate the relative importance of each predictor from variance decomposition (Grömping 2007). Statistics were computed in IBM-SPSS (v28.1) and significances were checked at *α* = 5%.

## Results

### Anthropometric, maturity, and training traits

Children’s characteristics, maturity status, and training times are given in Table 1a and b. Across follow-up, 17 children remained at TS I (pre-puberty), 12 passed from TS I to II, with 2 remaining at TS II (early puberty), and 13 passed from TS II–III to III–IV, with 2 remaining at TS IV (mid/late-puberty). Somatic maturity was earlier in girls (− 3.8 to + 0.9 years) than boys (− 4.8 to + 0.1 years). Starting age ranged from 3.5 to 10 years, while current training time ranged from 4.5 to 11 h/week at baseline and from 4 to 10.5 h/week at follow-up. Training time change in girls and boys was about similar at pre-puberty (≈ 170 h) and mid/late-puberty (≈ 210 h) and higher in boys (+ 45 h) at early puberty.

**Table 1:** (a) Girls’ and (b) boys’ characteristics per pubertal status group (mean ± sd, range)

	Pre-puberty		Early puberty		Mid/late-puberty	
	Baseline → follow-up		Baseline → follow-up^a^		Baseline → follow-up^b^	
(a)						
Age (year)	8.9 ± 1.1 (7.6–10.2)	9.8 ± 1.1 (8.5–11.1)	9.5 ± 1.1 (8.2–10.9)	10.3 ± 1.1 (9.0–11.7)	11.5 ± 0.9 (10.6–12.7)	12.4 ± 0.8 (11.5–13.5)
Height (cm)	129.9 ± 6.5	134.7 ± 6.9	138.6 ± 6.4	144.0 ± 7.1	154.7 ± 8.0	160.0 ± 6.5
Weight (kg)	27.2 ± 2.4	30.0 ± 2.5	32.5 ± 2.7	36.3 ± 3.1	44.6 ± 9.0	49.6 ± 8.6
APHV (years)	11.7 ± 0.3	11.9 ± 0.3	11.5 ± 0.5	11.7 ± 0.5	11.6 ± 0.2	11.7 ± 0.2
Maturity offset (years)	− 2.8 ± 0.8 (− 3.8 to − 1.8)	− 2.1 ± 0.9 (− 3.2 to − 1.1)	− 2.1 ± 0.8 (− 3.0 − 1.0)	− 1.4 ± 0.8 (− 2.3 to − 0.2)	− 0.1 ± 1.0 (− 1.3 to 1.2)	0.7 ± 0.9 (− 0.6 to 1.9)
Tanner stage I/II/III/IV (sample size)	5/0/0/0	5/0/0/0	7/0/0/0	0/7/0/0	0/5/4/0	0/0/5/4
Starting age (year) (2–3 h/week)	4.3 ± 0.7 (4.0–5.5)		4.9 ± 0.6 (4.0–5.5)		5.4 ± 1.4 (3.5–8.0)	
Weekly training (h/week)	5.8 ± 1.2 (4.5–7.5)	6.4 ± 1.5 (4.5–7.5)	6.6 ± 1.8 (5.0–9.0)	7.2 ± 2.2 (4.0–10.0)	8.1 ± 1.5 (6.0–11.0)	8.1 ± 1.4 (6.0–10.5)
Training Time (h)	514 ± 246 (228–774)	683 ± 292 (348– 999)	535 ± 130 (436–710)	722 ± 186 (472–958)	937 ± 333 (454–1554)	1156 ± 366 (634–1848)
(b)						
Age (years)	9.0 ± 0.8 (7.5–10.4)	9.8 ± 0.8 (8.3–11.3)	10.3 ± 0.8 (9.5–11.8)	11.2 ± 0.8 (10.3–12.6)	11.5 ± 1.3 (9.7–13.0)	12.4 ± 1.3 (10.6–13.8)
Height (cm)	133.6 ± 6.0	138.4 ± 6.7	139.9 ± 5.4	144.1 ± 5.2	151.5 ± 6.1	158.0 ± 7.2
Weight (kg)	34.1 ± 6.2	37.5 ± 6.6	35.6 ± 3.2	37.9 ± 3.5	43.8 ± 8.2	49.2 ± 9.7
APHV (years)	12.8 ± 0.5	13.1 ± 0.6	13.3 ± 0.6	13.7 ± 0.5	13.5 ± 0.4	13.5 ± 0.3
Maturity offset (years)	− 3.8 ± 0.5 (− 4.8 to − 2.6)	− 3.2 ± 0.5 (− 4.3 to − 2.1)	− 3.0 ± 0.4 (− 3.4 to − 2.4)	− 2.5 ± 0.5 (− 3.0 to − 1.8)	− 2.0 ± 0.9 (− 3.2 to − 0.8)	− 1.2 ± 1.1 (− 2.8–0.1)
Tanner stage I/II/III/IV (sample size)	12/0/0/0	12/0/0/0	5/2/0/0	0/7/0/0	0/2/2/2	0/0/2/4
Training history						
Starting age (years) (2–3 h/week)	5.3 ± 1.1 (3.5–7.0)		5.3 ± 1.0 (4.0–7.0)		6.6 ± 1.9 (4.0–10.0)	
Weekly training (h/week)	5.7 ± 0.9 (4.5–7.5)	6.5 ± 1.4 (4.0–9.0)	8.2 ± 2.8 (6.0–12.0)	8.4 ± 2.5 (6.0–12.5)	7.0 ± 1.8 (5.0–10.0)	7.4 ± 1.2 (6.0–9.5)
Training time (h)	473 ± 141 (136–728)	638 ± 169 (240–896)	648 ± 240 (400–1088)	880 ± 294 (610–1451)	736 ± 275 (424–1060)	942 ± 298 (568–1274)

^a^Two children remained at TS II for boys

^b^Two children remained at TS IV for boys

### BMC and BA per arm and asymmetry

**Fig. 1:**
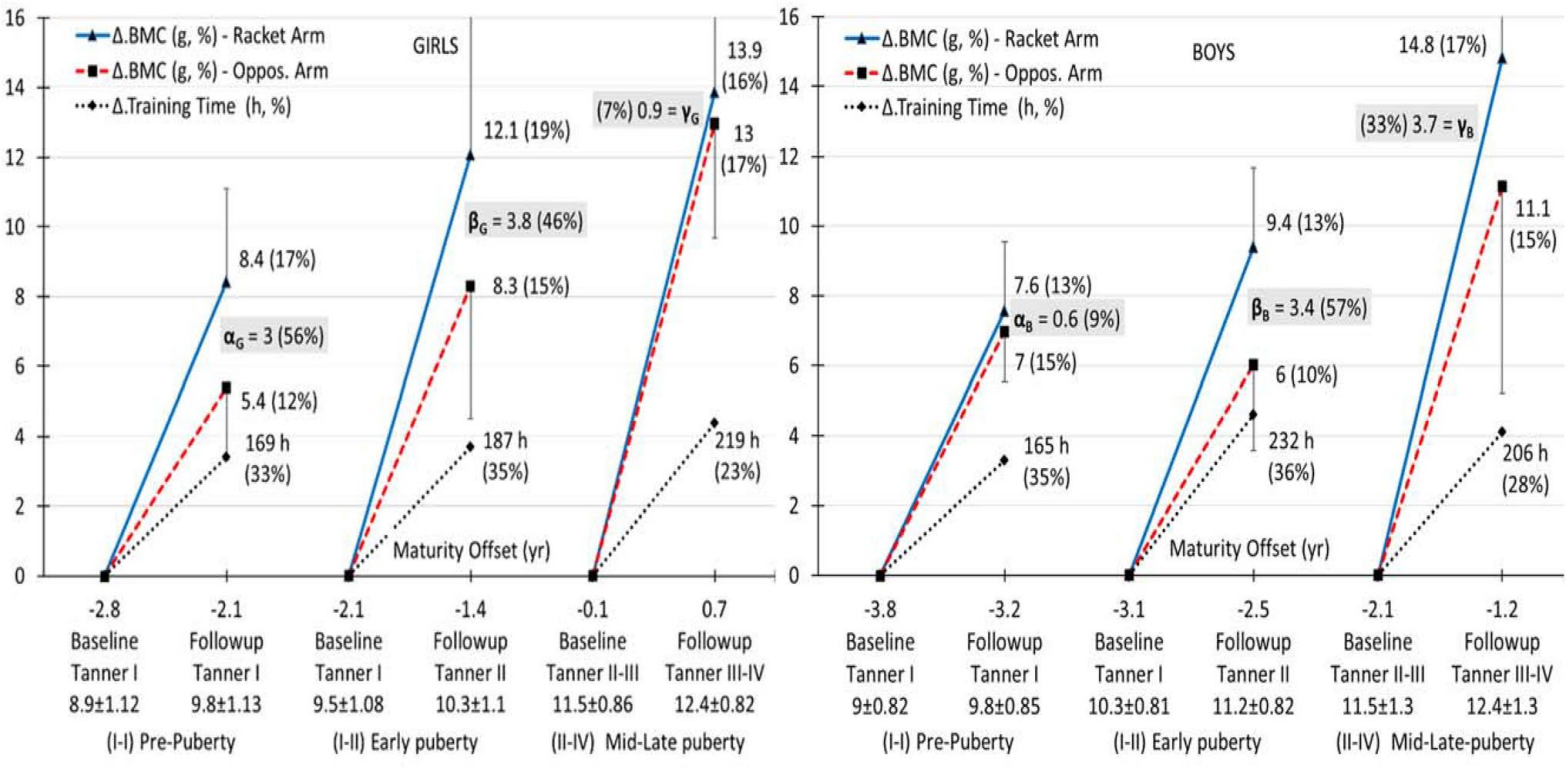
Change in BMC (mean, 95% CI) per arm and sex with change in training time (TT), maturity offset (MO), and tanner stage (TS) across age (for each change per arm *P* < 0.01, Cohen *d* > 0.8)

**Fig. 2:**
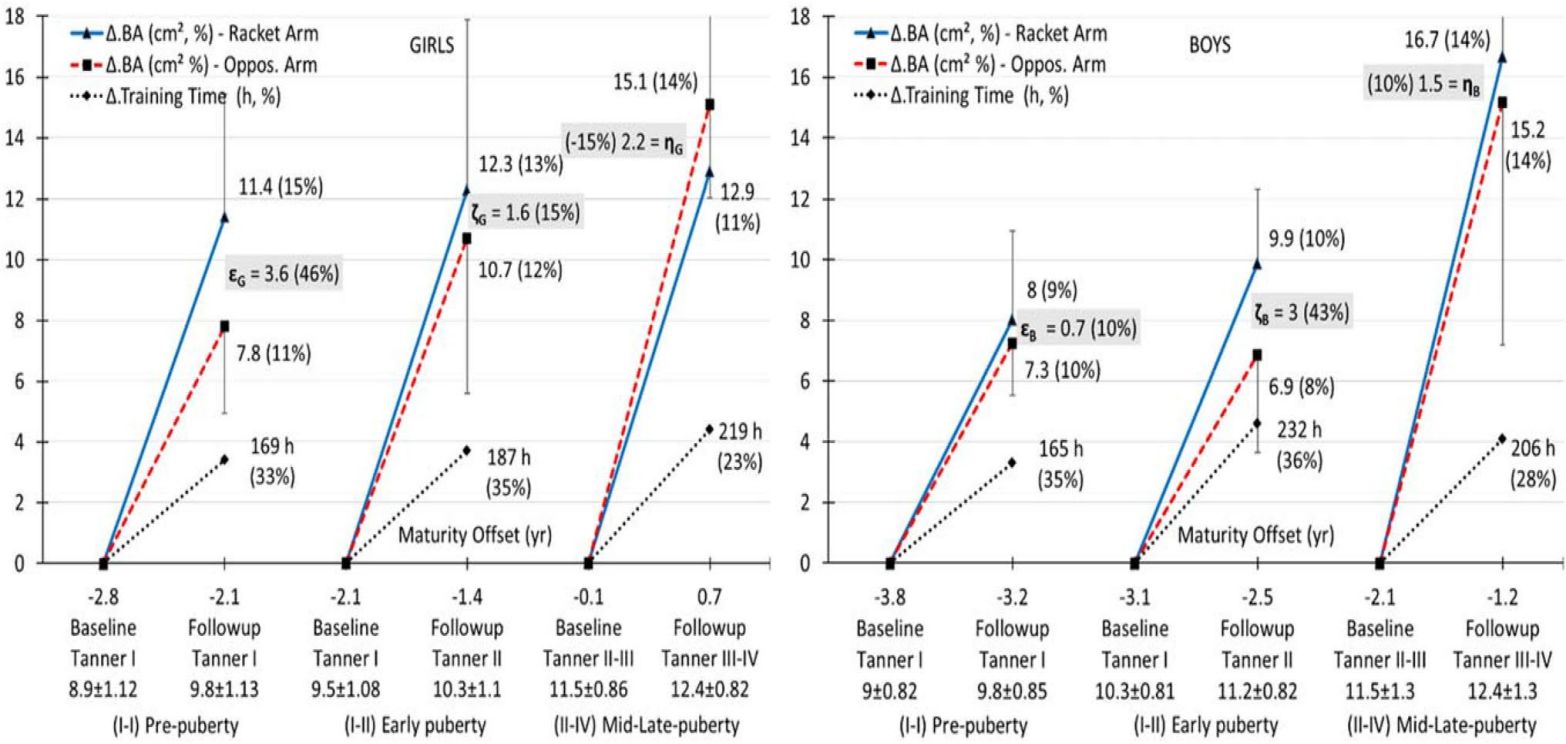
Change in BA (mean, 95% CI) per arm and sex with change in training time (TT), maturity offset (MO), and tanner stage (TS) across age (for each change per arm *P* < 0.01, Cohen *d* > 0.8)

Both bone parameters were significantly larger (*P* < 0.05, Cohen’s *d* > 1.0) in the RacArm than in the OppArm (asymmetry) in both sexes and pubertal groups (Tables 2 and 3). Across the three groups, % asymmetry changed in BMC by + 5, + 4, − 1.5 percentage points in girls and by − 2, + 3, + 3 percentage points in boys (Table 2); and in BA by + 4, + 2, − 3 in girls and by − 0.2, + 2, + 0.6 in boys, respectively (Table 3). Change (Δ) in BMC and BA per arm and group (Figs. 1 and 2) along MO (girls: − 2.8 to + 0.7 years; boys: − 3.8 to − 1.2 year) depict the osteogenic effect of exercise (RacArm) beyond growth (OppArm). Across groups and for both bone traits, this change was about linear in both arms and for both sexes, change in training hours was similar in both sexes, except early puberty, where boys accumulated a larger training time than girls (232 vs 187 h) and possessed the largest asymmetry change (57%).

**Table 2:** Bone mineral content (BMC, g) per arm, asymmetry, and pubertal status group for girls and boys (mean ± sd, 95% CI)

	Pre-puberty		Early puberty		Mid/late-puberty	
	Baseline → follow-up		Baseline → follow-up		Baseline → follow-up	
Girls						
BMC racket arm	49.7 ± 11.6	58.1 ± 13.2	61.7 ± 9.5	73.7 ± 15.5	100.2 ± 28.1	114.1 ± 26.2
BMC opposite arm	45.2 ± 8.4	50.6 ± 8.2	55.3 ± 9.7	63.6 ± 13.6	83.1 ± 22.5	96.0 ± 21.9
Asymmetry^a^ (95% CI)	4.5 ± 3.7 (− 0.1 to 9.0)	7.5 ± 5.3 (0.9–14.1)	6.4 ± 4.0 (2.7–10.1)	10.2 ± 5.3 (5.3–15.0)	17.2 ± 7.9 (11.1–23.2)	18.1 ± 7.0 (12.7–23.5)
% Asymmetry, prob., Cohen’s *d*	9.1 ± 6.9%, < 0.05, 1.2	13.9 ± 7.8%, < 0.01, 1.4	12.1 ± 8.1%, < 0.001, 1.6	16.4 ± 9.8%, < 0.001, 2.3	20.5 ± 7.2%, < 0.001, 2.2	18.9 ± 5.4%, < 0.001, 2.6
Boys						
BMC racket arm	58.9 ± 12.0	66.4 ± 12.9	71.1 ± 7.8	80.5 ± 8.7	90.4 ± 25.6	105.3 ± 28.5
BMC opposite arm	49.9 ± 11.4	56.9 ± 11.0	59.5 ± 6.0	65.5 ± 6.0	76.7 ± 23.9	87.9 ± 28.0
Asymmetry^a^ (95% CI)	9.0 ± 5.0 (− 0.1–9.0)	9.6 ± 6.9 (0.9–14.1)	11.6 ± 6.2 (2.7–10.1)	15.0 ± 5.1 (9.4–15.8)	13.7 ± 4.2 (9.0–16.3)	17.4 ± 6.2 (12.2–20.1)
% Asymmetry, prob., Cohen’s *d*	19.2 ± 12.6%, < 0.001, 1.9	17.4 ± 12.8%, < 0.001, 1.4	19.8 ± 10.4%, < 0.01, 1.6	22.9 ± 7.9%, < 0.001, 1.9	19.1 ± 7.2%, < 0.001, 3.2	22.0 ± 10.9%, < 0.001, 2.8

^a^Šidák-corrected α (set of 6 comparisons per arm) = 0.008

**Table 3:** Bone area (BA, cm^2^) per arm, asymmetry, and pubertal status group for girls and boys (mean ± sd, 95% CI)

	Pre-puberty		Early puberty		Mid/late-puberty	
	Baseline → follow-up		Baseline → follow-up		Baseline → follow-up	
Girls						
BA racket arm	76.8 ± 14.6	88.2 ± 14.6	96.1 ± 16.7	108.4 ± 19.6	133.3 ± 26.6	146.2 ± 23.1
BA opposite arm	71.8 ± 10.0	79.6 ± 11.4	88.0 ± 15.5	98.7 ± 19.2	118.7 ± 23.9	133.8 ± 22.0
Asymmetry^a^ (95% CI)	5.0 ± 5.1 (− 1.3–11.3)	8.6 ± 6.1 (1.0–16.2)	8.1 ± 4.1 (4.3–12.0)	9.7 ± 5.6 (4.6–14.9)	14.7 ± 5.8 (10.2–19.1)	12.4 ± 4.0 (9.4–15.5)
% Asymmetry, prob., Cohen’s *d*	6.4 ± 6.2%, < 0.05, 1.0	10.7 ± 6.6%, < 0.05, 1.4	9.4 ± 4.8%, < 0.001, 2.0	10.2 ± 6.3%, < 0.01, 1.7	12.5 ± 5.2%, < 0.001, 2.5	9.5 ± 2.9%, < 0.001, 3.1
Boys						
BA racket arm	87.6 ± 13.6	95.6 ± 13.9	101.9 ± 6.5	111.7 ± 7.5	119.5 ± 22.4	136.2 ± 25.0
BA opposite arm	78.7 ± 12.8	85.9 ± 13.0	90.0 ± 6.2	96.9 ± 5.4	108.0 ± 24.2	123.2 ± 29.4
Asymmetry^a^ (95% CI)	8.9 ± 5.8 (4.8–10.7)	9.7 ± 6.5 (6.2–12.5)	11.9 ± 3.0 (7.7–12.3)	14.9 ± 6.3 (8.7–16.0)	11.5 ± 4.5 (7.4–16.8)	13.0 ± 8.5 (7.5–18.2)
% Asymmetry, prob., Cohen’s *d*	11.8 ± 8.5%, < 0.001, 1.5	11.6 ± 7.9%, < 0.001, 1.4	13.3 ± 3.7%, < 0.001, 3.9	15.4 ± 6.6%, < 0.001, 2.4	11.5 ± 5.8%, < 0.001, 2.6	12.1 ± 9.5%, < 0.01, 1.5

^a^Šidák-corrected *α* (set of 6 comparisons per arm) = 0.008

In BMC, asymmetry change was large (> 33%) and ranged from about 3–4 g for girls and boys, but at pre- (*α*) to early puberty (*β*) for the first and at early (*β*) to mid/late-puberty (*γ*) for the latter; sex difference (*P* > 0.05) was moderate (*α*, *d* = 0.62), small (*β*, *d* = 0.11), and moderate to large (*γ*, *d* = 0.74) for the three pubertal groups, respectively (Fig. 1). In BA, asymmetry change was large (> 43%) and ranged from about 3–4 cm^2^ for girls and boys, but at pre-puberty (*ε*) for the first and at early puberty (*ζ*) for the latter; sex difference (*P* > 0.05) was moderate (*ε*, *d* = 0.53), small (*ζ*, *d* = 0.26), and moderate (*η*, *d* = 0.66) for the three pubertal groups, respectively (Fig. 2).

### Predicting BMC and BA asymmetry change

The summary results of the regression of BMC and BA asymmetry change on the linear combination of sex and change in MO, TS, and TT (with baseline as the covariate) are given in Table 4. Both models were significant (*P* < 0.020) and non-collinear (tolerance > 0.70). Model 1 explained 28% of the variability in BMC asymmetry change; predictive importance was high for baseline asymmetry (38%) and TT change (41%). Model 2 explained 30% of the variability in BA asymmetry change; predictive importance was high for baseline asymmetry (58%) and moderate for MO change (17%) and TS change (12%). Sex was not impactful for both bone parameters, while TT was unimportant for BA asymmetry change.

**Table 4:** BMC and BA asymmetry change regressed on sex (girls *vs* boys), and change in maturity offset (MO), tanner stage (TS), and training time (TT), with baseline asymmetry as covariate

	(Model) dependent variable ($\hat{Y}$)							
	(1) BMC asymmetry change (g) [*F* = 3.05, *P* = 0.020, *R*^2^ = 0.28]				(2) BA asymmetry change (cm^2^) [*F* = 3.50, *P* = 0.010, *R*^2^ = 0.30]			
Predictor (*X*)	*b*	*P*	LMG	Toler	*b*	*P*	LMG	Toler
Constant	− 2.44	0.366		–	6.69	0.077		–
Sex (girls *vs* boys)	− 0.925	0.452	1.6%	0.74	− 1.511	0.366	4.4%	0.74
Baseline asymmetry	− 0.29	0.004	37.9%	0.73	− 0.51	< 0.001	58.4%	0.82
Maturity offset change (years)	1.50	0.663	2.5%	0.82	− 8.37	0.077	17.2%	0.81
Tanner stage change (0 vs 1)	1.99	0.111	16.9%	0.74	3.35	0.053	12%	0.71
Training time change (hours)	0.031	0.006	41.2%	0.74	0.020	0.142	8%	0.86

*b* standardized regression coefficient, *P* probability, *LMG* Shapley’s index of relative importance based on variance decomposition, *Toler.* tolerance (% non-collinearity)

## Discussion

This study showed that across 9 months of systematic tennis training the induced bone gains were maximized at pre-puberty in girls and at early puberty in boys, and then leveled-off or reduced at mid/late puberty in about the same manner for both sexes. This pattern of exercise-induced bone gains was reflected by the deviation of the trajectory of the RacArm from that of the OppArm in the three tested groups across MO change (Figs. 1 and 2). The OppArm follows its normal growth in about a linear fashion and about equally in boys and girls towards pubertal growth spurt (Mølgaard et al. 1997), which is associated with peak bone mass and size accretion at about the age of 13–14 years (Mølgaard et al. 1999). The distinction between mineralization and expansion across participation relates to the known time dissociation between bone mass and size (Mølgaard et al. 1999). In our data, the high relationship (0.73) between change in BMC and BA at pre-puberty (with 160–170 h of training) became moderate (0.44) at mid/late puberty, despite longer training times (200–220 h). Apparently, towards maturation bone adaptation to exercise tends to be less effective in size than in mass (Figs. 1 and 2). Even in longitudinal samples of boys and girls, peak bone expansion precedes peak mineralization (Faulkner et al. 2006). These osteogenic mass and size differentials occur around puberty. For example, in male tennis players 10–19 years humeral BA tends to plateau after TS III, despite longer training histories in the older players (Ducher et al. 2009), whereas even after 6 years of physical activity, children of about the same age reach their peak BMC accrual velocity about 1 year after APHV in both sexes (Bailey et al. 1999).

The pattern of asymmetry in the development of each arm in tennis players is more bone trait- than sex-specific, and roughly proportional to the training duration. In this respect our results for girls confirm those of Ducher et al. (2011) but not those of Chapelle et al. (2023). In girls at pre/peri puberty, the tennis-induced bone gains after 12 months of participation were higher than ours by about 2–3% points for the racket and opposite arm, respectively, in BMC and BA (Ducher et al. 2011). Therefore, the longer training duration induced a notable increase in the achieved bone gains. However, in Chapelle et al. (2023), the group of pre-, peri-, and post-pubertal girls showed about equal BMC gains to both arms for the first (15–16%) and the second (16%) year of participation; in their group of boys of about the same pubertal status, the respective BMC gains were lower compared to those of girls but and again equal to both arms (11–12%) per year of participation. Contrary, our BMC and BA asymmetries were substantial and about proportion to the accumulated training hours across 9 months of participation, with the disparities between boys and girls being more notable in the time pattern of bone change than on the magnitudes of the respective asymmetries (Figs. 1 and 2).

These sex-specific inconsistencies in bone mass and size growth with exercise are conditional on the synergistic effects of hormonal regulation which is unique to each stage of puberty (Bass 2000). The increased activity of GH, IGF-1, and sex steroids from pre- to peri-puberty leads to increased bone mineralization; the dichotomized androgenic and estrogenic effects regulate the differential timing of puberty and the final length of the skeleton in the two sexes, with a return of these hormones’ activity to pre-pubertal levels (Mauras et al. 1996). However, according to MacKelvie et al. (2002, Fig. 1, p. 251), in children involved in weight-bearing activities, the trajectories of estrogen and testosterone continue their slight gradual increase from pre- to early puberty, but then tend to slightly decline at about the age of 12 for GH and 13 for IGF-1 after peak BMC velocity. This asynchronous decline in GH and IGF-1 at TS III and IV explains partly the pattern of BMC and BA change in each arm at mid/late puberty (Figs. 1 and 2). Interestingly, for this stage of puberty, there was a BA asymmetry reversal in favor of the OppArm in girls (Fig. 2, *η*). Such exercise-associated bone width suppressions are paradoxical even for mature tennis players, which occasionally possess smaller cross-sectional periosteal and endocortical areas in their RacArm radius (Nara-Ashizawa et al. 2002). Our data for the mid/late-pubertal girls showed that at baseline the RacArm had about 14 cm^2^ higher BA than the OppArm, and it, thus, possessed a much lesser margin for further bone expansion. Evidently, towards the end of puberty girls reach their normal bone width and thus they possess a lesser margin for notable exercise-induced bone area gains. This may be partly linked to the notable decrease in the activity of bone cells towards late puberty (Mora et al. 1998), with bone formation in mass and density and bone resorption in bone volume (Mora et al. 1999).

Yet, as an inter-individual variation in the timing and tempo of sexual and somatic maturity in healthy children is obvious, there is always a need to separate the effects of training from those of maturity (Baxter-Jones et al. 2005). Thus, besides the comparative evaluation of the patterns of exercise-induced BMC and BA gains across puberty (Figs. 1 and 2), we also estimated the effects of training time after controlling for sexual maturity and somatic growth change (Table 4). This control for maturation was secured via separate modeling of BMC and BA, as the two measures express distinct bone traits and their link in children aged 6–18 years is rather curvilinear after adjusting for body size and puberty (Warner et al. 1998). Our regressions revealed the higher importance of training time than pubertal change for BMC and the moderately higher importance of somatic growth change than pubertal change for BA. As TT increases there is a large proportional enhancement of the bone mass, while as MO decreases there is a less proportional increase in bone expansion. Roughly, when TT increase by more than 25%, there is at least 40% gain in bone mass and at most 10% gain in bone area. These trends of bone gain with training volume occur mostly within the pre- to early puberty period inversely depended on the initial level of bone status (Ducher et al. 2011). When children start their training season with low levels of inter-arm bone asymmetries, they possess a higher margin to increase their bone gains (after an adequate amount of training hours) and vice versa. This inverse relationship (Table 4, negative *β* values for baseline asymmetry) was also observed by Ducher et al. (2009).

In this respect, it is obvious that exercise-induced bone gains tend to be maximized at pre- and early puberty, respectively, because early childhood is characterized by small inter-arm bone asymmetries. In fact, the covariances between physical activity and bone measures in children 4–6 years are only 2–9% (Janz et al. 2001), and this is indicative of the potential for better bone benefits when proper exercise starts even at early childhood. This potential can be better assessed if we know the degree of dependence of bone gain on exercise at distinct stages of childhood and puberty. In this case, it does not suffice to connect a training duration period of several months with the corresponding increases in grams of bone mass or in cm^2^ of bone area. Ducher et al. (2011) reported a 12-month training derived change in humeral asymmetry of 1.7 g (9.5%) in BMC and of 2.7 mm^2^ (30%) BA in pre/peri-pubertal girls 10–14 years. As an extension of these useful data, we approximated the amount of regular training time required to induce a threshold bone growth. Specifically, we estimated that for a bone mass gain of 1 g, the required training time is about 120 h at pre-puberty, and 60 h at early puberty, while for a bone width gain of 1 cm^2^, the required training time is about 100 h at pre-puberty, 90 h at early puberty, and 260 h at mid/late puberty.

**Fig. 3:**
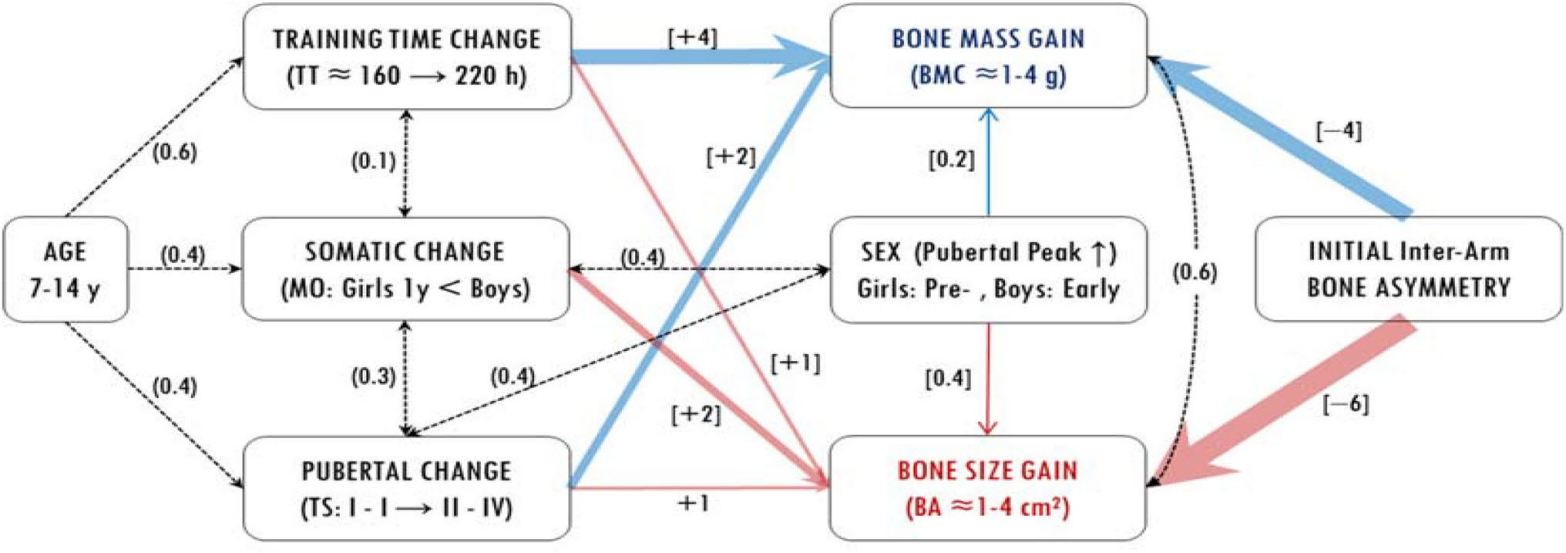
Flow chart of the relations and paths of influence between the factors of bone mass (BMC) and size (BA) gain; numbers in parenthesis show the correlations among the factors; numbers in brackets [1 to 10] and arrow thickness signify the relative contribution of the factors (negative signs show inverse correlations)

These bone gain analogies with exercise can be better appraised from the overview of the trends and flows of inter-actions among the major factors involved in the tennis-induced bone gains (Fig. 3). This overview combined data from our bone asymmetry estimates and from our overall regression testing. It thus clarified the patterns of influence directed from sex, training time, pubertal stage, and maturity offset on bone mass and size. In the absence of collinearities among these explanatory factors (Table 4), this novel approach allowed to determine the quantitative content of bone adaptation with exercise via variance decomposition (Grömping 2007). Age was included in this overview as it mediates bone adaptation across the progression of tennis activity (Ashizawa et al. 1999). Yet, due to its high collinearity with the other factors, age was omitted from the respective linear models to avoid redundancy. Thus, this flow chart allows for a more comprehensive reasoning of the phenomenon of osteogenesis with exercise across puberty.

In conclusion, a 9-month tennis participation by about 170–220 h of training induced substantial bone parameter and pubertal status specific gains in pre- and peri-pubertal children 7–14 years. Induced bone size gains occurred about one pubertal stage earlier than those of bone mass. Bone mass and size gains were maximized at pre-puberty in girls and at early puberty in boys. In both bone parameters and sexes, the exercise-induced bone gain tends to plateau or reduce at mid/late puberty, i.e., no further bone gains. The impact of exercise time was four times higher on bone mass than on bone size, and twice the impact of pubertal status on bone mass; the latter was equal (2/10) to the impact of somatic growth on bone size. The impact of sex was negligible on either bone trait. This osteogenesis depends largely on the state of bone asymmetries seen at baseline.

## Limitations and implications

Limitations are sample and design relevant. The technical skill of the children may have slightly limited the accuracy of our bone estimates, because playing techniques and neuromuscular coordination both improve from early childhood to the end of puberty. Therefore, children perform tennis serves with lesser muscular effort (Elliott et al. 2003) depending on equipment scaling (Farrow and Reid 2010). In addition, a control group of healthy children not involved in upper extremity unilateral sports was not feasible. Controls cannot be matched on heredity, nutrition, and lifestyle, nor on pre-existing handedness-derived bone asymmetries. Thus, the advantage of the internal control model of tennis was early recognized (Jones et al. 1977) and became common both in cross-sectional (Ducher et al. 2009; Ireland et al. 2013; Warden et al. 2021) and prospective studies (Chapelle et al. 2023; Ducher et al. 2011). Further, a larger sample would allow for more robust bone growth estimates and even separate regression fits per pubertal group. Yet, as noted in the methods, our sample size was almost adequate for testing these asymmetry estimates and for the two regressions fits according to common practices (Maxwell 2000; Pedhazur 2006). Lastly, the use of two-handed backhands may have limited our findings. However, two-handed backhands require less strength than one-handed backhands (Giangarra et al. 1993) and rely more on trunk rotation to accelerate the racket (Genevois et al. 2015); they are less effective on bone growth because they are less dynamic in terms of the mechanics of the interacting arm segments. Thus, this potential bias is rather small or non-existing in mature players (Ducher et al. 2005) and “confers no significant bone change” in youth players (Ireland et al. 2013). Similarly, pre-existing bone asymmetries due to handedness are rather small as seen in normal post adolescence (Pirnay et al. 1987), while handedness per se is assumed not to stimulate extra bone expansion in tennis players (Krahl et al. 1994). Biomechanically, both potential biases are assumed to exert counterbalancing effects on the asymmetry between the two arms.

Implications are growth relevant and stem from the established view that physical activity and sports should start at pre-puberty and be maintained through puberty to achieve maximal peak bone mass (Vicente-Rodríguez 2006). Therefore, tennis players would be given some extra training of the OppArm (Palaiothodorou et al. 2020) to gradually approach the level of bone strength of the RacArm, which achieves more than 10 years earlier the protective advantage of “prevention of bone loss and osteoporotic fractures” (Kannus et al. 1994). The observed time dissociation between bone mass and size by about one TS signifies an earlier bone expansion than mineralization, and this implies a period of “relative bone weakness” that characterizes about equally boys and girls (Faulkner et al. 2006). This aspect of bone growth should be under special attention by sport trainers, so that, even when performance is the sole purpose of participation, a progressive physical development of the children is adopted (Ochi and Campbell 2009). Thus, children should join training programs aiming to a “global body” growth as this would advance their technical and strength qualities before specialization, which demands intensive training (severely asymmetric in tennis) and has the risk of improvement reduction post-pubertally (Wiersma 2000). Our tennis-induced arm bone hypertrophy estimates were substantial even at the initial stage of pre-puberty, probably because most of these children started playing tennis some years earlier. Therefore, considering the scaling task constraints that emerge during children’s performance in racket sports in general (Fitzpatrick et al. 2018), it appears that there is some space for the foundation of bone enhancement even at early childhood.

## Data Availability

The datasets analyzed during the current study are available in the MENDELEY repository, Data, V1, 10.17632/2z8pzzs8m3.1.
